# Long-Term System Suitability Evaluation for Mass Accuracy in the Analysis of Small Molecules by High-Resolution Mass Spectrometry

**DOI:** 10.1021/jasms.5c00128

**Published:** 2025-08-13

**Authors:** Paul Löffler, Svante Rehnstam, Lutz Ahrens, Foon Yin Lai, Alberto Celma

**Affiliations:** Department of Aquatic Sciences and Assessment, 8095 Swedish University of Agricultural Sciences (SLU), Uppsala SE-75007, Sweden

**Keywords:** suspect screening, nontarget screening, quality assurance, Orbitrap, reproducibility, instrumental performance

## Abstract

High-resolution mass spectrometry (HRMS) is critical for the identification and characterization of (un)­known organic chemicals. In this regard, ensuring high mass accuracy in HRMS instruments is essential for reliable results in nontarget and suspect screening. This study presents a practical approach for evaluating and maintaining mass accuracy over time using ultrahigh pressure liquid chromatography coupled with electrospray ionization Orbitrap HRMS. A set of 13 reference standards, encompassing a range of polarities and chemical families, was analyzed before and after sample analysis batches to assess the impact of various factors on the instrumental performance regarding mass accuracy. The aim is not to recalibrate the system but to provide a reliable snapshot of the mass accuracy over time. The study found that the positive ionization mode exhibited higher accuracy and precision compared with the negative mode. Factors affecting mass accuracy included calibration quality, the number of batch injections, and the time between calibrations, where the two latter factors were related to each other. Results suggest that performing system suitability tests for high-resolution accurate masses with two injections before and after sample analysis is adequate for ensuring acceptable mass spectrometric performance for robust and reliable HRMS data acquisition, but performing three injections is recommended. This protocol ensures that informed decisions can be made with regard to the mass accuracy, the calibration, and a potential recalibration before HRMS data acquisition is performed.

## Graphical abstract

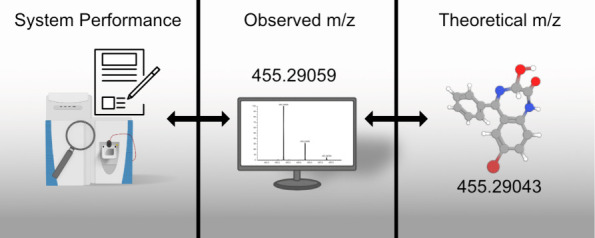

## 1 Introduction

The use of high-resolution mass spectrometry (HRMS) instrumentation for wide scope screening of organic micropollutants (OMPs) has significantly increased in recent years. This is due to the remarkable advancements in both hardware and software, as well as increased instrument robustness, which has greatly contributed to foster their utilization.^1–3^ Modern HRMS instruments are capable of providing high spectral resolving power and high mass accuracy, which are essential for the characterization of unknown compounds and the reliable identification of substances.^2,4^ The mass accuracy of an HRMS instrument refers to its ability to accurately determine the mass-to-charge ratio (*m*/*z*) of ions within a certain parts per million (ppm) or mDa range. Consequently, a good mass accuracy is crucial for the assignment of molecular formulas to observed *m*/*z* peaks that permits their subsequent identification/characterization.^2,5^ In brief, mass accuracy refers to the deviation of the measured mass from the true mass, wherein a good mass accuracy has an error below 3 ppm.^6^ Thus, a low deviation value (measured in ppm, or mDa) suggests a high level of accuracy.^4^

Poor mass accuracy in HRMS measurements will severely affect the data acquisition and processing.^2,3^ For instance, when analyzing samples in data-dependent acquisition mode, a high deviation in mass accuracy can result in the failure to determine ions that should undergo fragmentation and MS^2^ analysis and, therefore, generating false negative findings in the samples.^7^ Also, later during the data processing steps, a low mass accuracy will negatively impact the calculation of molecular formulas for both the intact molecule (precursor ion) and the fragments. Thus, the appropriate characterization of the molecules’ identity or structure may be hindered by the poor quality of the mass spectrometric measurements. Unfortunately, mass accuracy for HRMS instruments is affected by several instrumental and/or external factors such as time elapsed since last instrument calibration, instrument maintenance or changes in room temperature but also by sample-dependent factors such as matrix complexity or compound abundance.^2^ System suitability testing is a critical aspect for ensuring the accuracy and reliability of HRMS instruments.

Over the years, different strategies have been developed by manufacturers to ensure high mass accuracy in the measurement. For instance, time-of-flight mass analyzers usually implement on-the-fly mass correction based on the constant infusion of a well-known molecule (usually known as lock-mass), which aids in the correction of the mass analyzer deviation; or the recalibration of the mass axis every few seconds or injections in the instrumentation.^8^ However, the intrinsic characteristics of Orbitrap mass analyzers make it a highly stable instrument that does not experience strong drift during short spans of time. A simple calibration is often enough to ensure mass accuracies with a mass error below 3 ppm.^9^ Thus, the development and implementation of mass accuracy evaluation strategies for HRMS studies based on Orbitrap mass analyzers are pivotal in obtaining meaningful and reliable data. It is important to note that a successful calibration also does not always guarantee a high-quality calibration, as the instrument, in this case a QExactive Focus Orbitrap, may accept a calibration even with TIC variations far above the vendor’s recommendation. This further implies that the system’s suitability to analyze real samples needs to be evaluated in an after-calibration-before-analysis approach.

In this work, we have developed, implemented, and evaluated a strategy to assess the robustness of the mass accuracy in an ultrahigh-pressure liquid chromatograph (UHPLC) coupled to an Orbitrap HRMS instrument. In this sense, a set of reference standards have been regularly analyzed both before and after sample batches to study the impact of different parameters and/or factors on the instrument’s performance. We then evaluated the impact of time elapsed since last instrument’s calibration, the length of the acquisition batch, and other parameters such as the quality of the performed calibration. Based on the gathered data, we propose a system suitability check-up strategy to implement in HRMS screening analyses to ensure reliability of the generated data and results for target, suspect, and nontarget screening analysis of small molecules. This High-Resolution Accurate Mass-System Suitability Test (HRAM-SST) strategy is designed to serve as an indicative assessment of mass accuracy only and not as a calibration procedure. It does not result in a full system suitability test, such as controlling signal intensities or consistent retention times, but rather only for an empirical confirmation of system readiness for obtaining high-resolution accurate masses.

## 2 Materials and methods

### 2.1 Chemicals and Solution Preparation

Thirteen different chemicals were selected for use in our in-house high-resolution accurate mass system suitability test (HRAM-SST) for HRMS mass accuracy. These compounds were selected to cover both positive (POS+) and negative (NEG−) ionization modes as well as a wide range of *m*/*z*, a range of polarities, chemical families and functional groups. Compound stability was also key for the selection of chemicals to include in the HRAM-SST strategy. The compounds decided on considering the research group’s research interests, the *m*/*z* range of interest, and availability. It is recommended that other research groups seeking to make their own system suitability tests approach their compound selection in a similar way. Especially important is the idea that the solution is diverse, to assess if the instrument can perform in a diverse chemical space, has reliable and reproducible results across different chemical groups, and can give reliable accurate masses no matter the compound.^10^ Detailed information about the selected compounds can be found in Table 1. For the ease of understanding and text flow, from now on, all *m*/*z* values will be referred to in the text as nominal values.

**Table 1:** Compounds Used for the HRAM-SST

Compound Name (nominal mass)	Molecular Formula	Log *K*_OW_ ^a^	Adduct	Expected *m*/*z*	PubChem CID	CAS number
Acetaminophen (150 and 152)	C_8_H_9_NO_2_	0.46	–H	150.0561	1983	103-90-2
			+H	152.0706		
Anhydro erythromycin (716)	C_37_H_65_NO_12_	3.2	+H	716.4580	83949	23893-13-2
Caffeine (195)	C_8_H_10_N_4_O_2_	–0.07	+H	195.0877	2519	58-08-2
Carbamazepine (237)	C_15_H_12_N_2_O	2.45	+H	237.1022	2554	294-46-4
Clindamycin sulfoxide (441)	C_18_H_33_ClN_2_O_6_S	1.1	+H	441.1821	73046007	22341-46-5
Fexofenadine (502)	C_32_H_39_NO_4_	3	+H	502.2952	3348	83799-24-0
6:2 Fluorotelomer sulfonic acid (427)	C_8_H_5_F_13_O_3_S	3.9	–H	426.9679	119688	27619-97-2
Oxazepam (287)	C_15_H_11_ClN_2_O_2_	2.24	+H	287.0582	4616	604-75-1
Perfluorohexanoic acid (312)	C_6_HF_11_O_2_	3.6	–H	312.9728	67542	307-24-4
Perfluorooctane sulfonamide (498)	C_8_H_2_F_17_NO_2_S	5.8	–H	497.9462	69785	754-91-6
Perfluorooctanoic acid (413)	C_8_HF_15_O_2_	6.3	–H	412.9664	9554	335-67-1
Perfluoropentanoic acid (263)	C_6_HF_9_O_2_	2.9	–H	262.9760	75921	2706-90-3
Verapamil (455)	C_27_H_38_N_2_O_4_	2.15	+H	455.2904	2520	52-53-9

a Octanol–water partition coefficient (*K*_OW_).

A stock mixture solution of the HRAM-SST compounds at 2.5 μg/mL was prepared in methanol and stored at −20 °C. From that, a working solution at 50 ng/mL in methanol was prepared for each injection in the HRMS Orbitrap system. The working solution was prepared in 100% organic solvent to avoid the degradation of potentially water sensitive chemicals such as clindamycin sulfoxide or anhydro erythromycin.

For details of the contents of the calibration solutions see the Instrumentation section and Table S1 and Table S4 in the Supporting Information.

### 2.2 High-Resolution Accurate Mass System Suitability Test Strategy

This HRAM-SST approach is not intended to replace the manufacturer calibration routines. Instead, it offers a complementary indicative check of mass accuracy using representative compounds before and after sample analysis. As a routine for HRMS users, an evaluation of the performance of the instruments before and after their acquisition batches was conducted. In this sense, the HRAM-SST was injected onto the system using the generic analysis method explained in the following section but using the same chromatographic column and mobile phases that the analyst needed for their respective method. It should be noted that the precise chemical composition of the mobile phases was not systematic across all injections as the different analysts utilized the mobile phases they would later on use in their real samples analysis. Since the overall aim is not to obtain ideal and robust separation but to measure mass accuracy, it was deemed unnecessary to optimize the chromatographic separation and let the HRMS users utilize their own mobile phases composition for the ease of the HRAM-SST approach application.

Both positive and negative ionization modes had to be evaluated in quintuplicate (*n* = 5) per ionization mode. Thus, each acquisition batch (defined as one set of samples run on the instrument, usually within 2 and 136 injections each) corresponds to a data set for HRAM-SST evaluation of five replicates, each in positive preanalysis, negative preanalysis, positive postanalysis, and negative postanalysis. However, on a limited number of occasions, analysts ran HRAM-SST only in the negative ion mode. In total, 445 HRAM-SST injections were gathered for the evaluation.

After HRAM-SST injections, extracted ion chromatograms (EICs) of each compound were inspected using Freestyle (version 1.8.51.0, Thermo Scientific, Bremen, Germany) where *m*/*z* was taken from the apex of the peak. The observed *m*/*z* was compared to the theoretical *m*/*z* where the criterion for an instrument that is suitable for analysis was set to a maximum mass deviation of 3 ppm as well as a second criterion that the deviations should be randomly distributed (avoiding positive or negative biases).^9,10^ All *m*/*z* values were manually input into a digital spreadsheet where mass accuracies in ppm were calculated. If disagreement with the criterion happened, analysts recalibrated the instrument and reanalyzed the HRAM-SST. The evaluated mass accuracies were then recorded and used as the data set for this study.

### 2.3 Instrumentation

Instrumental analysis was performed using a Vanquish Horizon UHPLC instrument coupled to a QExactive Focus Orbitrap mass spectrometer (Thermo Fisher Scientific, Bremen, Germany) using an Ion Max heated electrospray ionization source (HESI-II) in both POS+ and NEG– ionization modes. In brief, 10 μL of a standard working solution was injected into the respective chromatographic column and separated with a generic and short gradient. At first, mobile phase A (generally consisting of Milli-Q water with or without modifiers, depending on analysts’ needs for their own runs) was held at 95% for 0.5 min to then linearly change to 5% over 4.5 min. After 4.5 min, mobile phase B (generally consisting of MeOH or ACN with or without modifiers, depending on analysts’ needs for their own runs) was held at 95% for 0.5 min. Finally, mobile composition came back at 95% A and 5% B for 1 min for re-equilibration of the system. Detailed composition of mobiles phases with the used modifiers can be found in Table S5 and Figure S1 in the Supporting Information.

For ion generation, the ion source parameters were set as outlined in Table S6. In POS+, the sheath gas flow rate was 35, the auxiliary gas flow rate was 10, and the spray voltage was set to 3 kV. In NEG–, the sheath gas flow rate was 45, the auxiliary gas flow rate remained at 10, and the spray voltage was set at −2.7 kV. The capillary temperature was set at 350 °C in positive and negative ionization mode. The auxiliary gas heater temperature was set at 300 °C in positive and 400 °C in negative ionization mode. Mass spectrometric analysis was performed with a resolution of 70,000 at *m*/*z* 200. The scan range was set from *m*/*z* 100 to 1000 in the full scan function.

Instrument calibration was performed following routines recommended by Thermo Fisher Scientific by means of calibration solutions Pierce LTQ Velos ESI Positive Ion Calibration Solution, Pierce Negative Ion Calibration Solution, from now on referred as to “CalMix” solutions and Pierce FlexMix (referred as ‘FlexMix‘) Calibration Solution from Thermo Fisher Scientific (Bremen, Germany). More information about the calibrants and their *m*/*z* can be found in Tables S3–S5 in the Supporting Information.

### 2.4 Statistical Analysis

All statistical analysis was done using R (version 4.3.1) with “data.table”, “magrittr” and “ggplot2” as main libraries. To explore the influence of various parameters on the instrument’s performance, we applied linear regression analysis. For each individual mass, we analyzed the mass error using a linear model with time (before and after the batch), number of injections, and the interaction of the injections as predictors. Additionally, the impact of calibration on the absolute mass error was assessed using another linear model. This model included calibration type (CalMix or CalMix + FlexMix), calibration quality, and time since the last calibration as predictors. A threshold for calibration quality was established at a root-mean-square (rms) of 0.3 ppm error as recommended by Thermo Fisher Scientific^11^ for both standard (CalMix) and custom calibrations (CalMix + FlexMix), with values below this threshold considered indicative for good calibration. In the linear model, the interaction between time since the last calibration and calibration quality was considered. However, due to sparse data on CalMix-only calibrations with long intervals since the last calibration, these cases were excluded from the analysis to ensure the robustness and reliability of the model’s results. The data and code can be found here: https://github.com/paloeffler/HRMS_SST.

## 3 Results and Discussion

### 3.1 Performance of the HRAM-SST Compounds

The mean and standard deviation for the mass accuracy of the HRAM-SST compounds over a five-month period, after conducting a total of 445 injections, are detailed in Table 2. This table compares the average mass error and corresponding standard deviation of the errors for both polarities using data from all five HRAM-SST injection replicates in every batch. Additionally, with the aim of exploring the efficiency of SST with a minimum number of replicates for a meaningful interpretation of the HRAM-SST results, data from only the first two injections are also included for comparison.

**Table 2:** Average Mass Error and Standard Deviation Per Investigated Mass for 5 and 2 Injections before and after the Samples^a^

Polarity	Nominal mass [Da]	5 injections		2 injections	
		Mean mass error [ppm]	Standard deviation [ppm]	Mean mass error [ppm]	Standard deviation [ppm]
+	152	0.310	0.757	0.313	0.727
	195	0.069	0.783	0.100	0.737
	237	0.402	0.729	0.467	0.681
	287	–0.003	0.716	0.018	0.677
	441	–0.190	0.708	–0.147	0.652
	455	0.127	0.736	0.161	0.674
	502	–0.127	0.738	–0.097	0.734
	716	–0.015	0.748	0.022	0.657
**+**	**Average/Standard deviation**	**0.072**	**0.024**	**0.105**	**0.035**
-	262	0.942	0.548	0.960	0.533
	312	–0.884	0.479	–0.873	0.48
	412	–0.008	0.555	–0.031	0.547
	426	0.761	0.613	0.748	0.623
	497	–0.046	0.498	–0.053	0.460
-	**Average/Standard deviation**	**0.153**	**0.053**	**0.150**	**0.063**

a Additionally, overall average mass deviation and standard deviation by polarity (including all HRAM-SST compounds evaluated) over a five-month HRAM-SST period. For the 2 injection assessment, the first 2 injections of the HRAM-SST set were considered.

For NEG–, the mean mass error with 5 injections before and after batch remained consistent with that of only 2 injections before and after batch, although there was a slight increase in standard deviation. This increase in variation was deemed low enough to not affect the instrument’s performance evaluation (Table 2, Figure 1). In contrast, POS+ results showed increases in both the mean mass error and its standard deviation when comparing the HRAM-SST strategy with 5 or 2 injections, although all values remained within the acceptable 3 ppm threshold. In general, the POS+ mass error showed both a lower mean value (0.072 ppm) and a lower standard deviation (0.024 ppm) compared to negative polarity (0.153 ± 0.053 ppm), indicating higher accuracy and precision in positive ionization mode as well as robustness against the many factors that can affect mass accuracy over time. Similar observations were pointed out during instrument calibrations, when the mass error of calibrants revealed a notably tighter distribution in positive polarity compared to the broader spread in negative polarity (Figure S2). Figure 1 also indicates that the median values of the averaged mass errors per nominal mass in POS+ have less variation across the different HRAM-SST compounds compared to those in NEG– (with the 25th–75th percentile interval ranging from −0.5 to 1 in POS+ and from −1.5 to 1.5 in NEG−). Although the median errors are generally centered around 0 in POS+, the broader distribution of median mass errors were observed in NEG–. This remains unexplained by the current data. Despite the wider distribution, the standard deviations of mass accuracy for each individual HRAM-SST compound in NEG– are significantly smaller than those in POS+ over the course of the 445 injections spanning five months of routine instrument use (*p* < 0.05).

**Figure 1:**
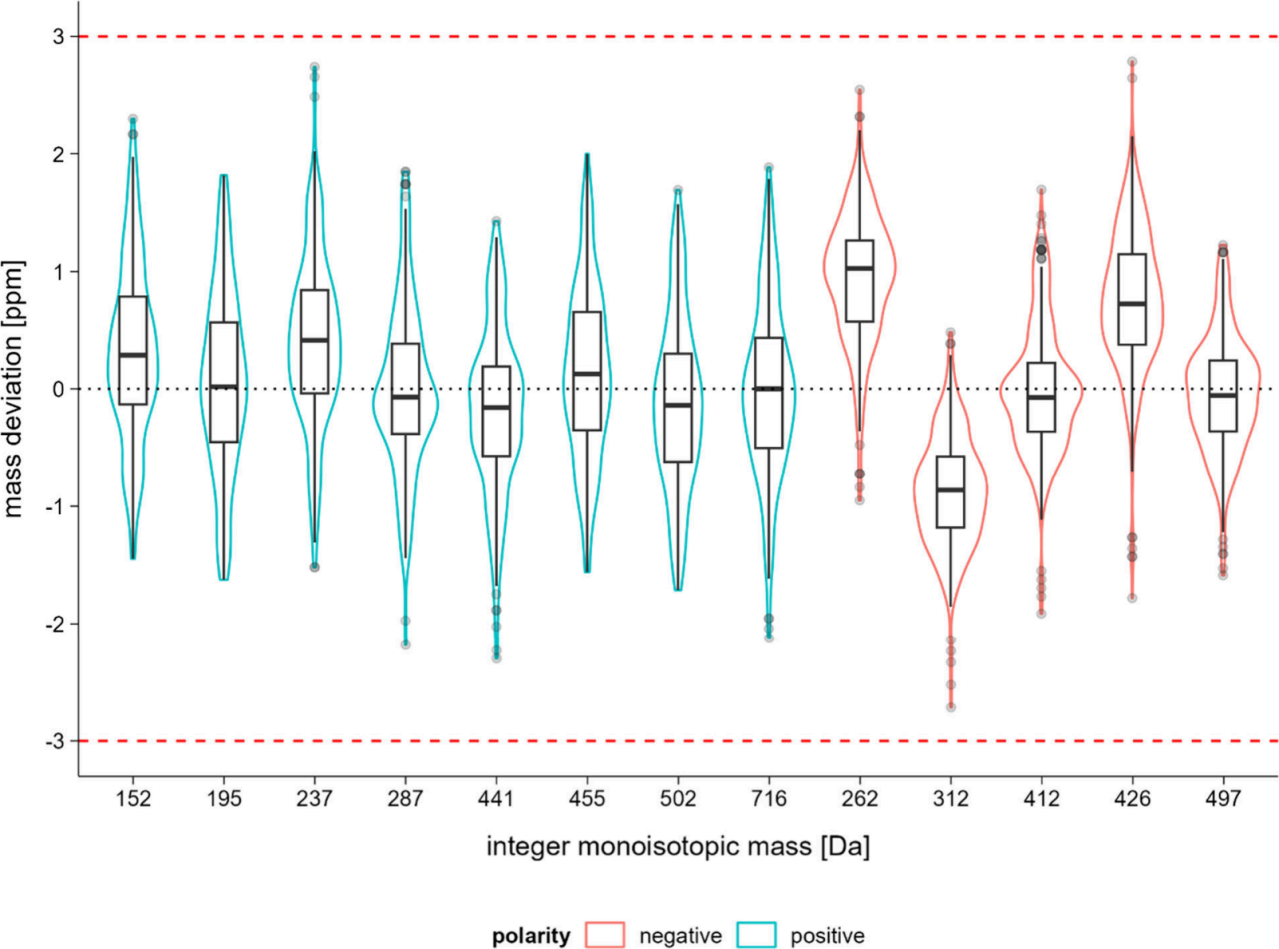
Mass error per investigated nominal mass for positive and negative mode, based on five injections before/after analyzed samples (*n* = 445). Median is displayed as a line and red dotted lines as 3 ppm threshold.

### 3.2 Impact of Nonmass Spectrometric Parameters on Mass Accuracy

When plotting the mass errors for each of the individual masses within the HRAM-SST mixture against the date of acquisition, no visual trend was observed regarding the influence of different columns, additives, or users (Figures S1, S3 and S4). Therein, the error distribution is most likely random, and no correlation with any of such parameters can be derived. Thus, given the low variance observed for the column type and additives, these variables were considered to have near-zero variance and were excluded from further analysis.

When performing the statistical analysis, the number of batch injections was used a measurement to indicate how long a batch was ongoing. We estimate that an average analysis in our lab, corresponding to one injection, is roughly 20 min long. Therefore, one injection will from now one be referred to as 20 min, and larger numbers converted to hours and/or minutes. The amount of time that has passed since the analysis started (i.e., the number of real sample injections performed between successive HRAM-SST analyses) significantly influenced the mass accuracy in POS+, with a strong negative effect observed as the number of injections increased (*p* = 2.75 × 10^–11^). Specifically, longer time frames correlated with a worse mass accuracy. However, for most compounds (*m*/*z* 152, 237, 287, 441, 455, 716), this effect was not significant when time passed was less than roughly 33 h (hours) (less than 100 injections). For example, in the case of *m*/*z* 195, a slight significant influence of batch injections persisted even with <33 h (100 injections) (*p* = 0.0417, linear model). When larger batches (>33 h) were included, the effect became more pronounced and significant (*p* = 0.006), suggesting a consistent trend. Conversely, *m*/*z* 502 showed no significant influence of time passed on mass accuracy, regardless of the amount of time since the analysis started (*p* > 0.05). The results overall indicate that the majority of HRAM-SST compounds were affected by the passing of time, highlighting that when the analysis exceeds 33 h (100 injections), a HRAM-SST analysis should be performed for ensuring reliable mass accuracy.

In NEG–, no consistent trend was observed across most of the compounds. Three compounds (*m*/*z* 262, 412, and 497) exhibited a relationship between mass accuracy and the time passed when batch sizes exceeded 16–20 h (50–60 injections), with improved accuracy observed in smaller batches. It was also observed that *m*/*z* 312 showed inconsistent variation in mass accuracy based on time passed. For *m*/*z* 426, no significant impact of batch injections or their interaction with time passed was observed when all batches were included (*p* > 0.05). However, when the analysis was limited to below 33 h, a significant influence of time passed (*p* = 6.92 × 10^–6^) and their interaction with time (*p* = 0.000889) was observed. Even an Orbitrap, which is known for having low drift, will experience a decrease in mass accuracy over time. Drift is mainly caused by external magnetic fields or shifts in ambient temperature, which will cause the mass accuracy to deteriorate over time.^9^ This suggests that the effect of batch injections on mass accuracy increases between HRAM-SSTs performed before versus after a long batch. These findings further emphasize the importance of limiting the run time to below 33 h (∼100 injections) to reduce variability in mass accuracy, as after 33 h (∼100 injections), a HRAM-SST analysis is recommended. Notably, the low *R* ^2^ values in the linear regression models (typically around ∼0.06) indicate that the statistical models explain only a small proportion of the variation in mass deviation. The complexity of the factors influencing mass accuracy suggests that additional unmeasured variables may contribute to the observed variability. While the identified trends, such as the effect of batch injections, remain valid and statistically significant, they account for only part of the variability in the data. These findings underline the importance of cautious interpretation, as the models provide insights into general patterns but do not fully capture the system’s complexity. Practical recommendations, such as limiting run time to less than 33 h (∼100 injections), are based on these observed trends and aim to optimize the mass accuracy. A simple drift analysis based on HRAM-SST deviation before and after each batch confirmed that mass accuracy can deteriorate with longer batch runtimes, even with good calibrations (Figure S5). Therefore, routine HRAM-SST checks are recommended after ∼100 injections of continuous operation. However, it is essential to recognize that the system’s inherent variability means these guidelines should be applied with consideration of specific experimental conditions and other influencing factors.

Regarding the influence of the type of calibration, all linear models of each mass showed a significant intercept, meaning that even immediately after good CalMix and FlexMix calibration of the instrument, a mass error (of the absolute mean value) significantly different from zero (*p* < 2 × 10^–16^) can be expected. However, this mass error was within the 2 ppm range (Figure 2, Figure S6), which still falls within the widely accepted 3 ppm for mass accuracy in most of the HRMS studies.^6,12,13^ On the contrary, higher errors were observed using NEG–, which is in line with our findings (Figure 1, Figure S6). Previous findings in scientific literature found that NEG– is less stable than POS+.^14,15^

**Figure 2:**
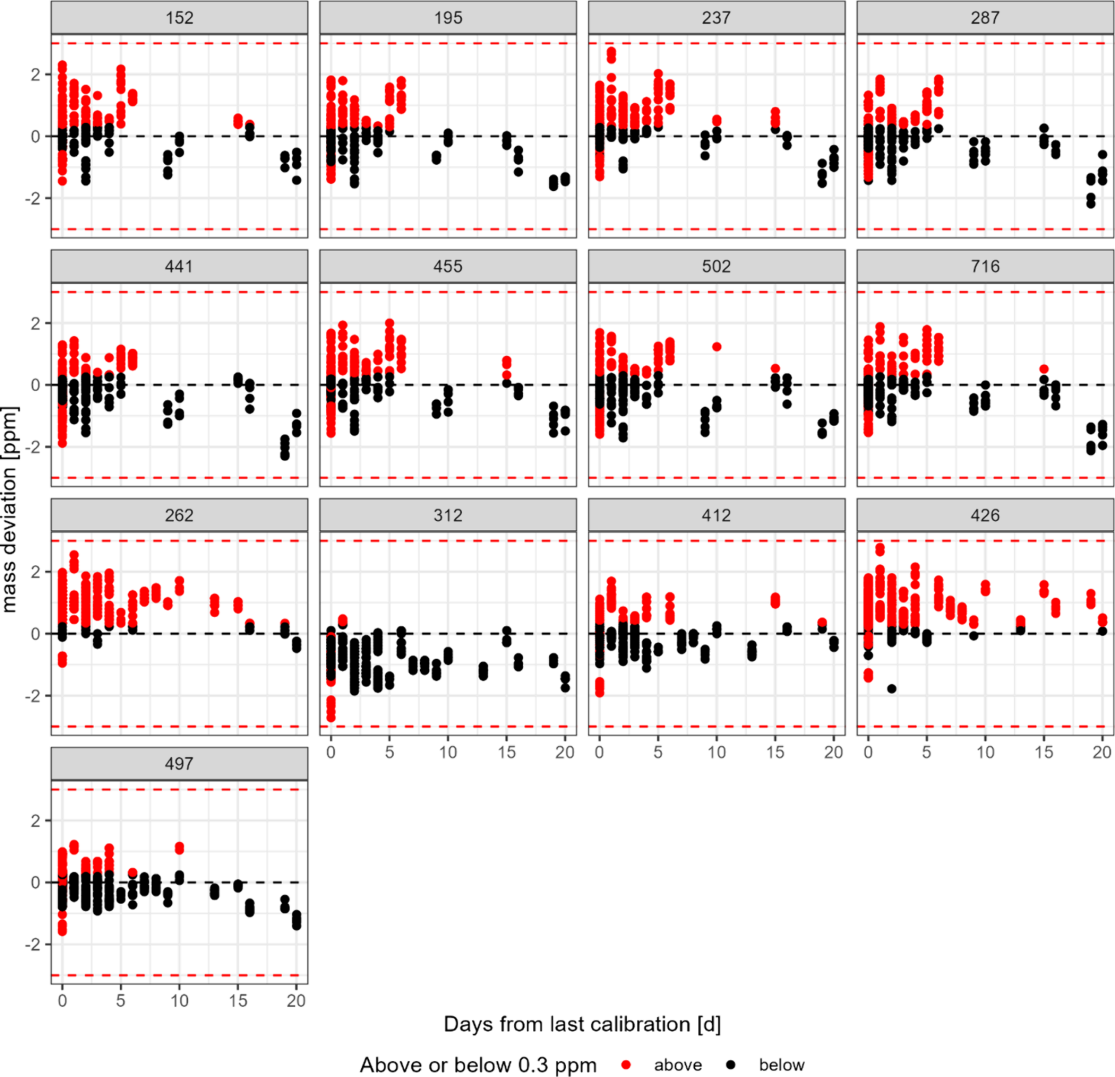
Distance of last calibration (days, in *x*-axis) displayed against mass deviation with 0 ppm error displayed as a dashed black line and 3 ppm threshold as a dashed red line (*y*-axis) (*n* = 445). Calibrations with quality values above the selected criteria of a rms of 0.3 ppm were colored in red, with values below this threshold (black dots) considered indicative for good calibration.

For the assessment of mass accuracy for calibrations with poor quality (consisting of accepting calibrations with quality over 0.3 ppm rms error for both internal and external calibration) almost all masses in positive mode (*m*/*z* 195, 237, 287, 441, 502, 716) showed significant mass accuracy changes (*p* < 0.05). Only *m*/*z* 152 (*p* = 0.43) and *m*/*z* 455 (*p* = 0.08) were not affected. In contrast, in negative mode, only the *m*/*z* 312 was barely significantly affected by a bad calibration (*p* = 0.04). While all the investigated compounds have *m*/*z* values within the calibrated range for the mass axis and, thus, the instrument should be working with the highest mass accuracy,^16^ an unreliable calibration can heavily impact the quality of the data gathered, resulting in unreliable data and as such cannot be used for in depth interpretation of Orbitrap behavior. It is important to highlight that the calibration did not at any point “fail”, nor was it deemed by the instrument software to be insufficient. It is only our own judgment that deemed a calibration to have poor quality. The lack of a clear quality evaluation of the calibration may cause an unexperienced Orbitrap user to believe they have appropriately calibrated the instrument and then proceed to acquire poor quality data. This would waste time, solvent, and sample, which is easily avoided by running a HRAM-SST.

Further investigation of the impact of calibration was done for the HRAM-SST approach. Orbitrap HRMS instruments are usually calibrated only with the CalMix calibration as suggested by the manufacturer.^17^ However, when small molecule analysis is intended, an additional customized mass calibration in the lower *m*/*z* range can be performed (FlexMix calibration). Data showed that using only CalMix calibration instead of CalMix + FlexMix often increased the mass error for seven of the eight investigated masses in POS+ from 0.1 ppm to up to 0.6 ppm (Table S4, Figure S6). In the example of caffeine, when calibrated using only CalMix (which lacks low *m*/*z* calibrants), the average mass deviation was 1.03 ppm. In contrast, when FlexMix (which includes low *m*/*z* calibrants) was added, the mass deviation improved to 0.42 ppm under a good calibration quality (Table S4). Performing a calibration with masses that are in proximity of the masses of interest has proven to be essential when calibrating an Orbitrap.^9^ It is of high importance that the analysis range does not go outside the calibration range, as extrapolation in accurate mass measurements is undesirable. This highlights the importance of understanding the mass range of the calibrants in CalMix, where extending the calibrants to the appropriate mass range of interest improves the mass accuracy, as shown by HRAM-SST results.

Another relevant factor that can influence the mass accuracy of the HRMS instrument is the time that has elapsed since the last calibration. In this sense, it has been observed that the time since the last calibration (Figure 2) significantly affected the mass error for most compounds in positive mode (*m*/*z* 195, 287, 441, 455, 502, 716) and two in NEG– (*m*/*z* 312, 426) (*p* < 0.05). The interaction between time since calibration and poor calibration quality significantly worsened mass accuracy for 8 out of the 13 masses analyzed (*m*/*z* 152, 237, 287, 441, 262, 312, 426, 497) (*p* < 0.05).

Once the mass accuracy affecting factors have been identified, a cost and time efficient HRAM-SST strategy should be implemented for long-term mass accuracy and data reliability assurance. For this, we investigated whether 5 HRAM-SST injections were needed to ensure good instrument performance or if the number of injections could be lowered to 2 to increase the analysis throughput. As indicated in Table 2, average mass errors and the corresponding standard deviations were consistent even if only the initial 2 replicates out of the 5 injections were considered (Figure S7). This suggested that the minimum of two injections also provides satisfactory mass errors. Although both NEG– and POS+ showed reliable data from including only the first and second injections, we want to highlight that in case there is an outlier in any of these two injections, a third injection will become necessary. Therefore, following common scientific practices, which typically recommend a minimum of three replicates for improved reliability of data validation, we suggest performing three injections of the HRAM-SST standard mix before and after sample batch analysis. This approach optimizes operational efficiency without significantly extending the analysis duration one would have by performing five injections, thereby ensuring the reliability and precision of the HRMS data acquired.

### 3.3 Key Considerations for an HRAM-SST Strategy

This paper has evaluated the performance of the instrument with the aid of a HRAM-SST solution containing compounds that can ionize in POS+ and NEG–. In this section, we aim to summarize parts of the article in a way so that other scientists can develop their own HRAM-SST strategy. It is also important to note that this is not a calibration strategy nor will it improve the quality of your calibration. The overall goal of this procedure is to efficiently obtain a quality estimate of the instrument in use, which can inform the user if the instrument is ready for high-resolution mass measurements and if further calibration or adjustments are needed, and to document the system performance over time. We observed that for data sets with less optimal calibrations (Figure S5, “above 0.3”), mass accuracy drift can begin to increase after approximately 20 h of injections (≈60 injections, assuming 20 min/injection). This trend was less pronounced for the well-calibrated data. However, due to the limited number of bad calibration cases in our data set, there is some uncertainty regarding the precise time to significant drift. Therefore, we recommend performing a HRAM-SST both before and after analysis, to ensure high-quality mass accuracy is maintained throughout, especially since deviations may arise earlier than 33 h under suboptimal calibration conditions.

Compounds should be selected based on their stability so that a solution can be stored for an extended amount of time in an autosampler tray and kept at a controlled temperature without noticeable degradation. This is considered due to two different main reasons: reduce the amount of lab work by removing the need to use freshly made solutions and enable the solution to be left in the instrument to easily start an HRAM-SST analysis.

The mass range is an important aspect of the compounds, where they should fall with regular *m*/*z* intervals in the range. In the case of a 100 to 1000 *m*/*z* range, one compound per 100 to 200 *m*/*z* would be deemed ideal. This is to make sure that the calibration range is covered so that the mass accuracy is within the set limits and not performing poorly in any *m*/*z* ranges. When the HRAM-SST method was developed, the entire *m*/*z* range was not included; instead, the focus was on compounds that were similar in *m*/*z* to expected compounds, but it is recommended to follow the suggestions mentioned above.

Furthermore, the class of compounds may also be considered; however, we did not find it to be of particular high priority. The most important factor was that the compounds are known to the researchers using the instrument and that they are readily available in the lab. Their behavior should be predictable and known so that if any problems are observed when running the HRAM-SST, an experienced user can tell that something is wrong.

The compounds should also be relatively easily eluted with a variety of mobile phase and solid phase combinations to ensure that a generic method can elute all compounds without any fine-tuning. The method should be quick and simple, because the chromatography is not an important aspect of the HRAM-SST, which focuses on the mass accuracy.

## 4 Conclusions

In summary, this study has systematically assessed the system suitability of acquiring high-resolution accurate masses over time in an Orbitrap LC-HRMS instrument using a comprehensive set of 13 reference standards, exploring both POS+ and NEG– ionization modes. The findings reveal that POS+ consistently achieved higher accuracy and precision compared to NEG–, supporting the visual result of the calibration reports, which demonstrated tighter mass distribution in the POS+ and showcasing the reliability of the Orbitrap. Additional injections of a laboratory specific HRAM-SST mixture before and after each sample batch demonstrated the ability to ensure a mass accuracy within 2 ppm. Additionally, the implementation of both CalMix and FlexMix calibrations has been identified as critical for achieving optimal performance, further stabilizing mass errors across the analysis if small molecules are of interest. Statistical analysis highlighted the impact of the number of injections during a batch, with the effect vanishing for batches with a lower number than 100 injections. Furthermore, our study indicates that regular calibration plays a crucial role in maintaining instrument accuracy, particularly highlighted by the increased mass errors observed when extending the intervals between calibrations. The significant influence of calibration quality on mass accuracy strengthens the necessity for strict calibration schedules and protocols. Overall, the protocol developed in this study provides a reliable and indicative method for tracking mass accuracy in HRMS analysis and identifying potential performance issues in the instrument. By integrating three HRAM-SST injections and adhering to calibration routines, laboratories can keep high throughput while maintaining stringent mass accuracy quality standards. These findings can enhance the reliability of small molecule screening and identification tasks undertaken by using this analytical method.

## Supplementary Material



## Data Availability

The data and code for the statistical analysis can be found here: https://github.com/paloeffler/HRMS_SST.

## Notes

Data on calibrants and compounds used, additional information on the influence of calibration performance, instrumental details, impact of mobile phase additives, chromatographic column impact, user, run time and time elapsed since last calibration impact on mass accuracy (PDF)
