# Supplementary material for: Long-Term System Suitability Evaluation for Mass Accuracy in the Analysis of Small Molecules by High-Resolution Mass Spectrometry
Source: J Am Soc Mass Spectrom. 2025 Aug 13;36(9):2005–12. doi: 10.1021/jasms.5c00128 (PMC12412142; doi:10.1021/jasms.5c00128)
Supplement: Supplementary file 1 [file js5c00128_si_001.pdf]

Supplementary information

Long-term system suitability evaluation for mass accuracy in the analysis of small molecules by high-resolution mass spectrometry

Paul Löffler*, Svante Rehnstam*, Lutz Ahrens, Foon Yin Lai, Alberto Celma[#]

Department of Aquatic Sciences and Assessment, Swedish University of Agricultural Sciences (SLU), Uppsala, SE-75007, Sweden

* Co-first author

[#] Corresponding author. Alberto Celma; email: alberto.celma.tirado@slu.se

Table S1: Calibrants for positive ionization mode (CalMix solution).

Compound	m/z
Caffeine	195.0877
MRFA 2+	262.6359
	393.2242
MRFA 1+	524.2646
Ultramark 1621	1022.0034
	1122.997
	1221.9906
	1321.9842
	1421.9778
	1521.9714
	1621.9650
	1721.9586
	1821.9522

Table S2: Calibrants for negative ionization mode (CalMix solution).

Compound	m/z
Sodium Dodecyl Sulfate	265.1479
Sodium taurocholate	514.2849
Ultramark 1621	1233.9742
	1279.9980
	1333.9678
	1379.9916
	1433.9614
	1479.9853
	1533.9550
	1579.9787
	1633.9486
	1679.9726
	1733.9422
	1786.1122
	1886.1056

Table S3: Compounds included in the FlexMix solution

**Compounds**	**Positive Ion. Mode (*m/z*)**	**Negative ion. Mode (*m/z*)**
Acetic acid	-	59.0128
Imidazole	69.0447	-
Triethylamine	102.1277	-
Trifluoroacetic acid	-	112.9845
Tetramethylpiperidine	142.1590	-
Pentafluoropropionic acid	-	162.9818
Caffeine	195.0877	-
Hexamethoxyphophazene	322.0481	-
Perfluorohexanoic acid	-	362.9696
MRFA	524.2649	-
2,4,6-tris(heptafluoropropyl)-1,3,5-triazine	-	601.9779
Hexakis(2,2-difluoroethoxy)phosphazene	622.0289	--
Hexakis(2,2,3,3-tetrafluoropropoxy)phosphazene	922.0098	1033.9870
Ultramark 1621	1022.0034	1233.9742
	1121.9970	1333.9678
	1221.9906	1433.9614
	1321.9842	1533.9550
	1421.9778	1633.9486
	1521.9714	1733.9422
	1621.9650	1833.9358
	1721.9586	1933.9294
	1821.9522	2033.9230
	1921.9458	2133.9166
	2021.9394	-
Hexakis(1h,1h,7h-perfluoroheptoxy)phosphazene	2121.9331	2233.9104
Hexakis(1h,1h,9h-perfluorononyloxy)phosphazene	2721.8948	2833.8720

Table S4: Influence of calibration on mass deviation and significance of trend analysis

Mass [Da]	Accepted calibration using CalMix + FlexMix	Poor calibration using CalMix + FlexMix		Calibration using CalMix only	
	Mass deviation [ppm]	p-value[a]	mass deviation [ppm]	p-value[a]	mass deviation [ppm]
152	0.55	>0.05	0.55	<0.001	0.98
195	0.42	0.01	0.62	<0.001	1.03
237	0.61	0.03	0.41	0.07	-0.2
287	0.36	0.04	0.51	<0.01	0.6
441	0.35	<0.001	0.78	<0.01	0.54
455	0.45	>0.05	0.45	<0.001	0.86
502	0.42	<0.001	0.74	<0.01	0.69
716	0.37	0.01	0.57	<0.001	0.74
262	1.09	>0.05	1.09	<0.001	0.46
312	0.84	0.04	0.98	<0.05	0.67
412	0.34	>0.05	0.34	<0.001	0.63
426	0.90	>0.05	0.9	<0.05	1.08
497	0.36	>0.05	0.36	<0.02	0.46

[a] significant test is based on t-test on the regression coefficients within each linear model.

Table S5: Lists of all columns and mobile phase additives used for SST acquisition. All additives were added to both the aqueous and the organic solvents of the mobile phase.

Column	Mobile phase additive
Acquity C18	5 mM acetic acid
Acquity C18	0.1 % formic acid
Acquity C18	0.01% formic acid and 5 mM ammonium formate
Acquity C18	0.1% ammonium hydroxide
Acquity C18	1% formic acid
Acquity C18	0.01M pyrrolidine
Cortechs C18	0.1% formic acid
Cortechs C18	5 mM acetic acid
Biphenyl 1.7	0.1% formic acid
EVO C18	5 mM acetic acid

Table S6: Ion source parameters used during the system suitability tests (SST) data acquisition

Parameter	POS(+)	NEG(-)
Sheath gas flow rate	35	45
Aux gas flow rate	10	10
Sweep gas flow rate	0	0
Spray voltage (kV)	3	-2.7
Capillary temp. (°C)	350	350
S-lens RF level	55	25
Aux gas heater temp (°C)	300	400

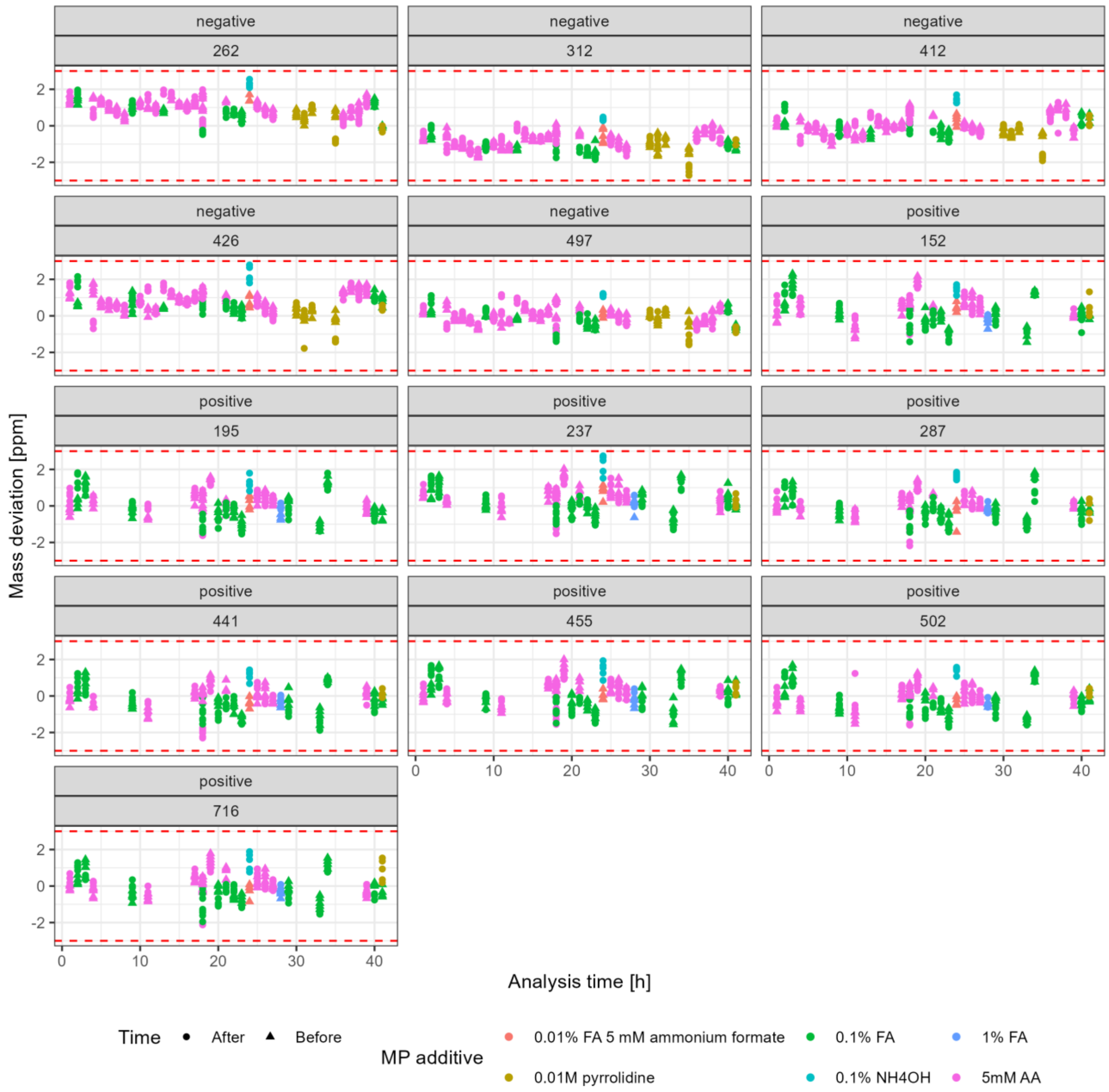

Figure S1: Mobile phase (MP) additives of the different batches, as proxy for time. Triangular points represent the SST injections before the batch and circular ones the injections after the respective batch in negative and positive mode using different mobile phase (MP) additives.

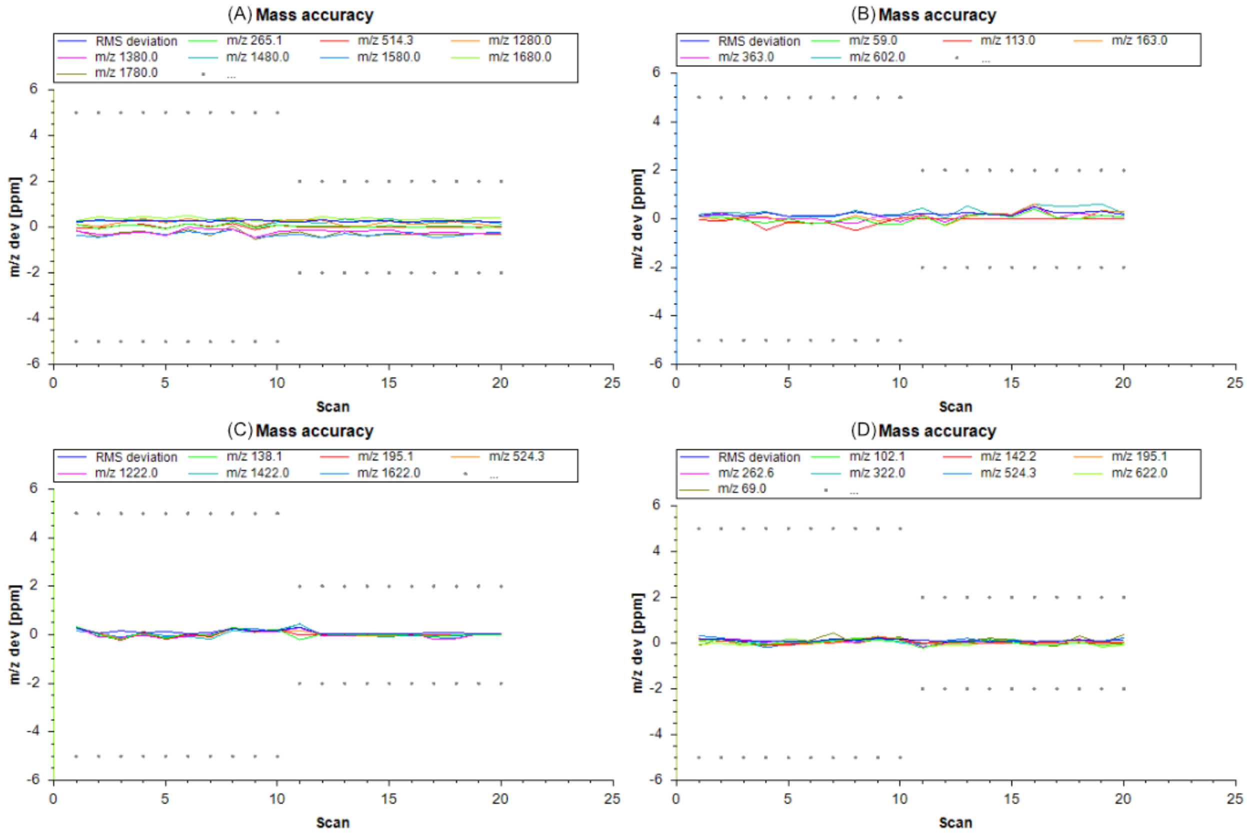

Figure S2: Calibration spectras from mass calibration of negative mode using CalMix (A), mass calibration in negative mode using FlexMix (B), mass calibration in positive mode using CalMix (C), mass calibration in positive mode using FlexMix (D).

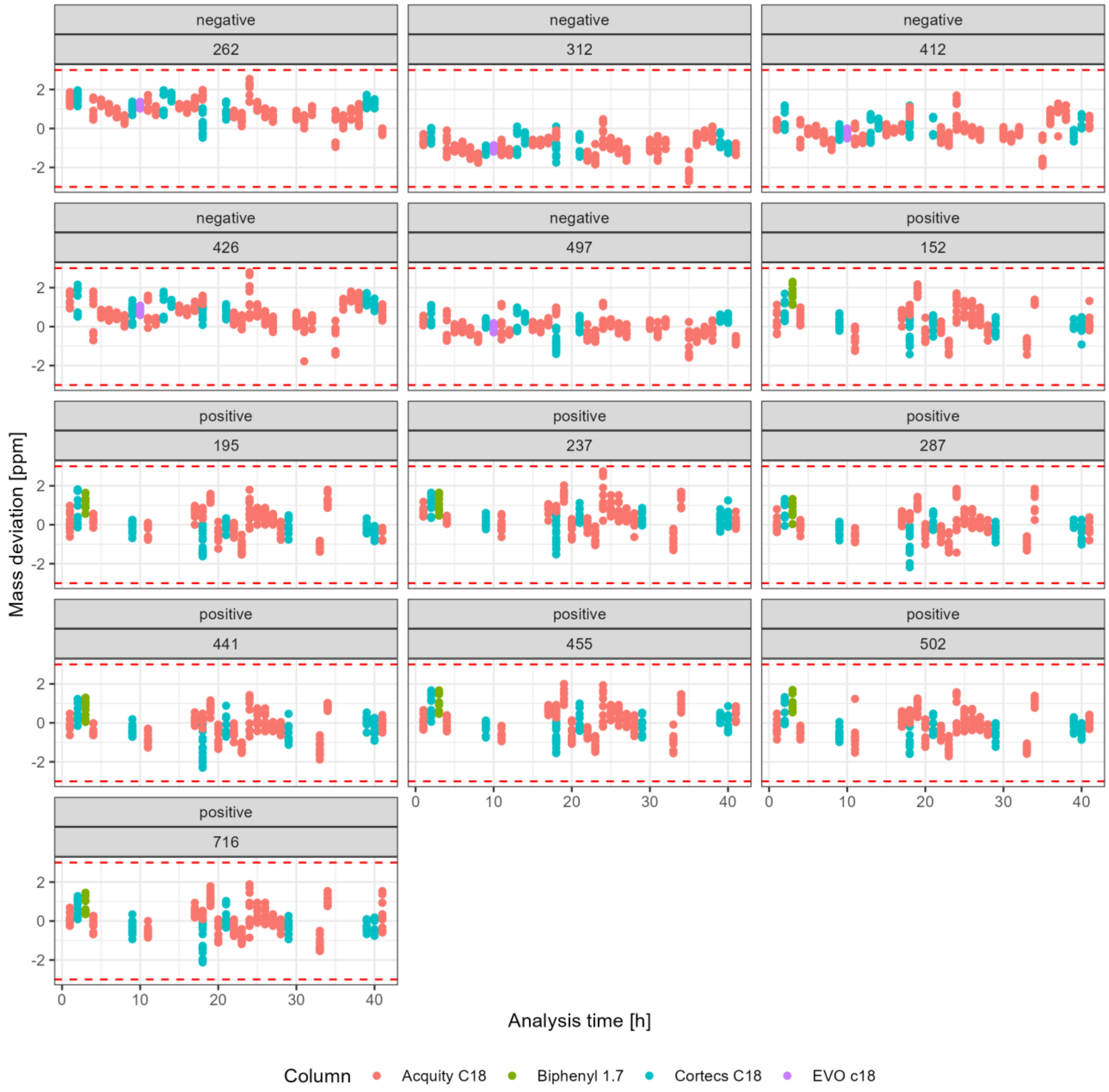

Figure S3: SST results showing mass deviation (ppm) of different chromatographic columns against number of batches injected as a reference of time.

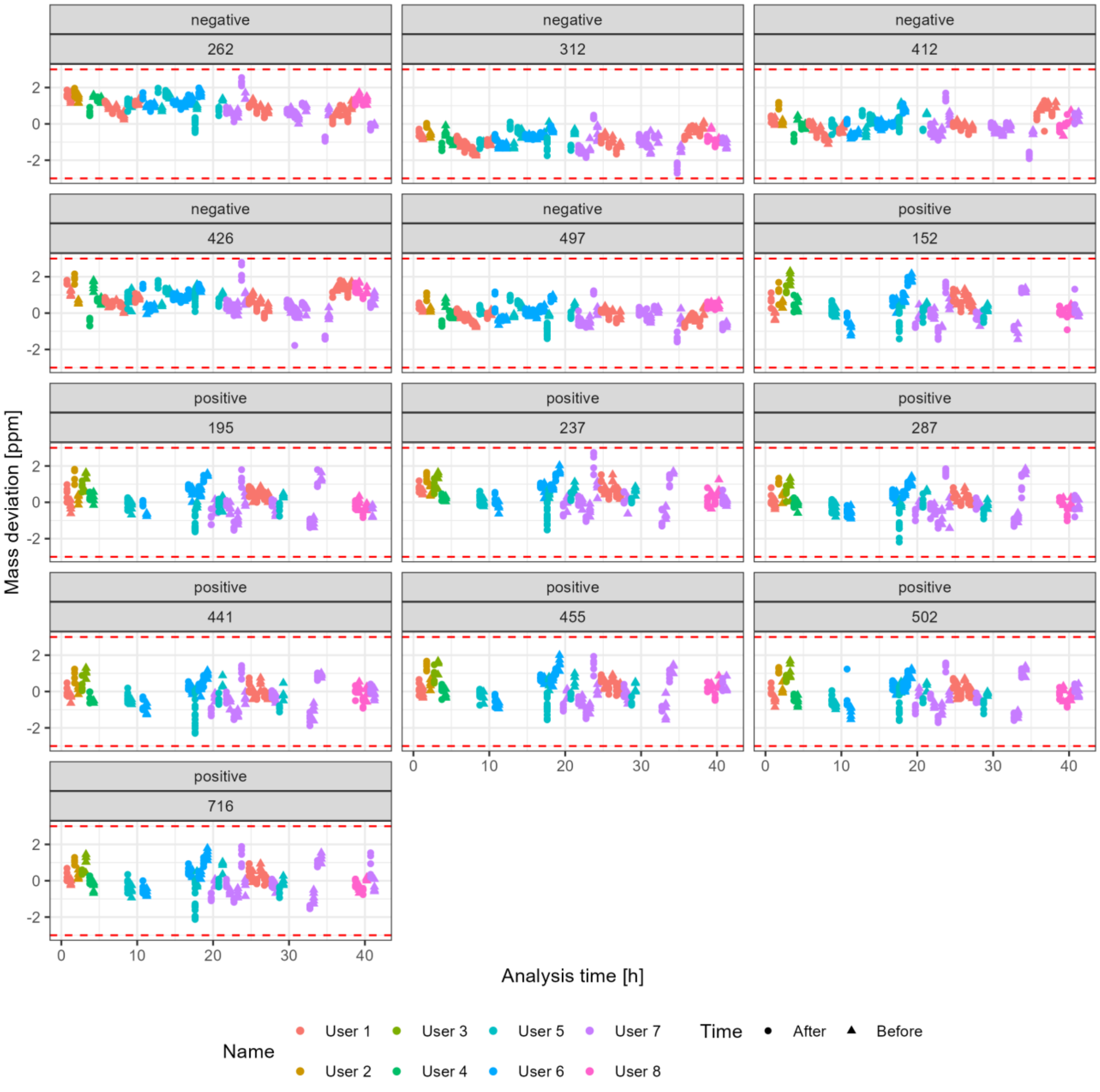

Figure S4: Mass deviation of the SST injections colored by the respective user of the Orbitrap. Symbol again symbolizing the injection time of the SST (before and after the respective batch).

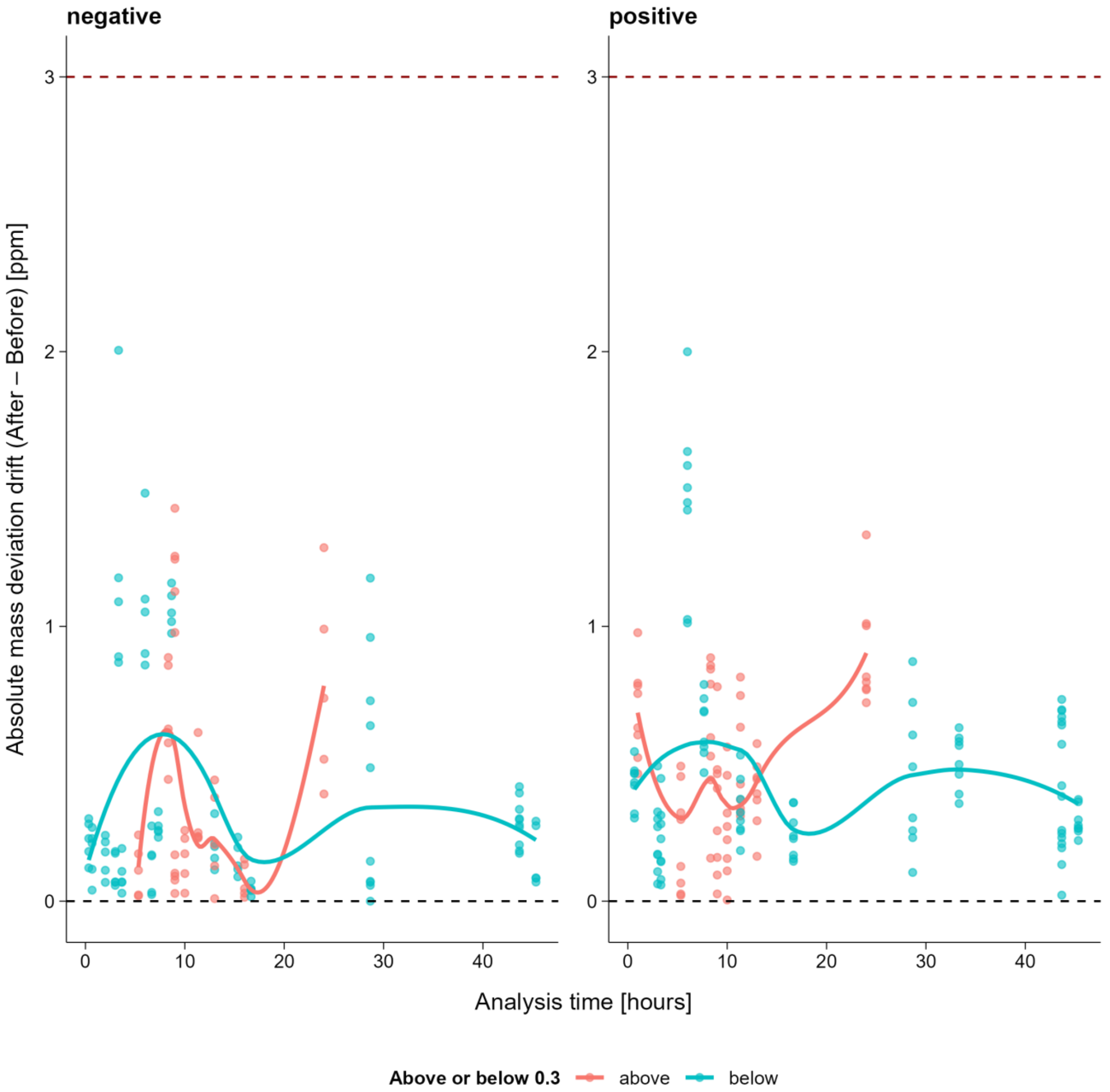

Figure S5: Absolute mass accuracy drift (After − Before) plotted against estimated number of injections per batch as proxy for runtime for both polarities. Each point represents a batch, and point colors indicate calibration quality (good or poor). A smoothed trend line (loess) is shown to highlight general drift behavior over time.

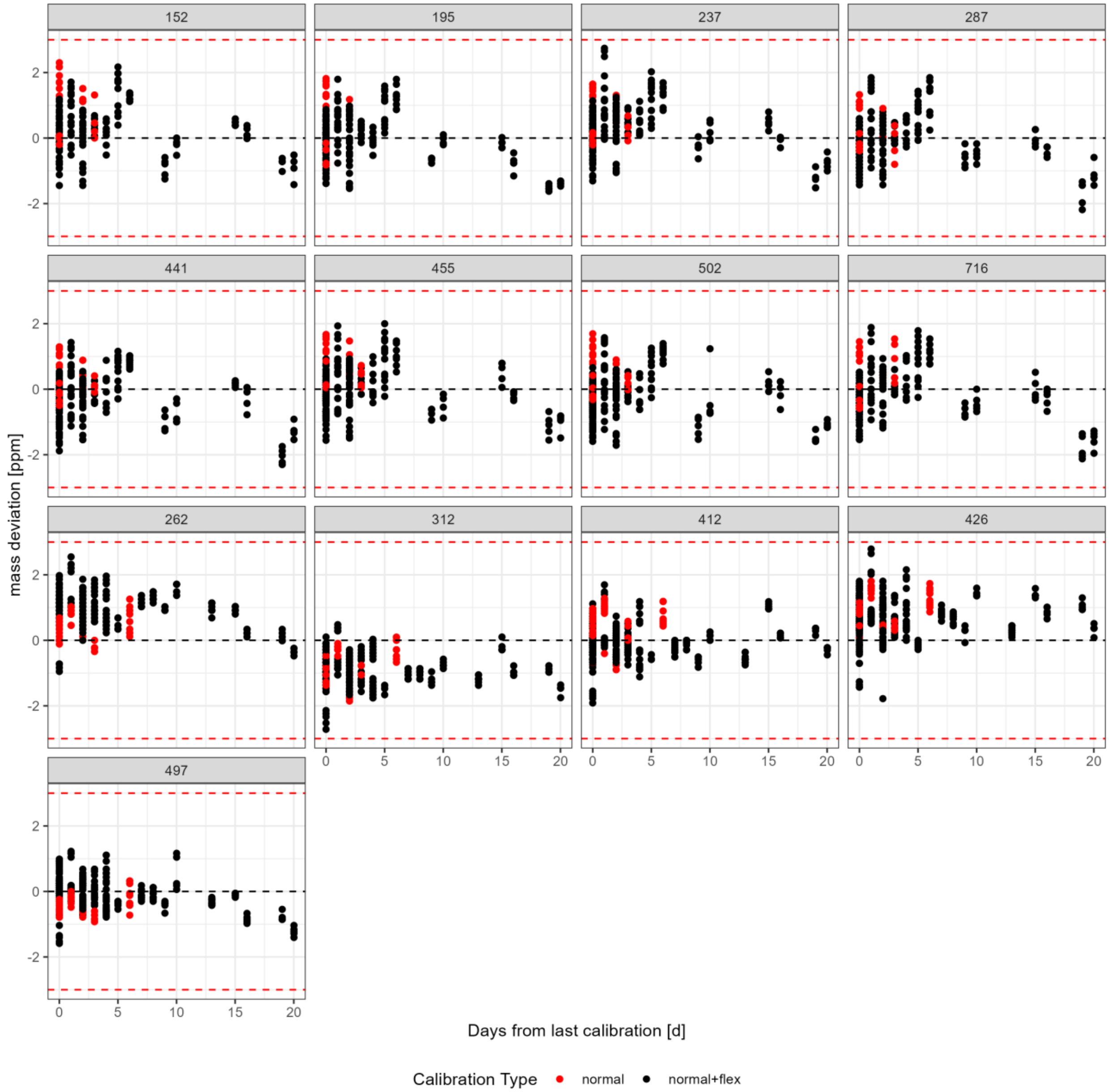

Figure S6: Distance of last calibration against mass deviation of SST compounds, colored by the different types of calibration. Red dots correspond to a calibration only performed with CalMix, black dots correspond to a calibration performed with both CalMix and FlexMix.

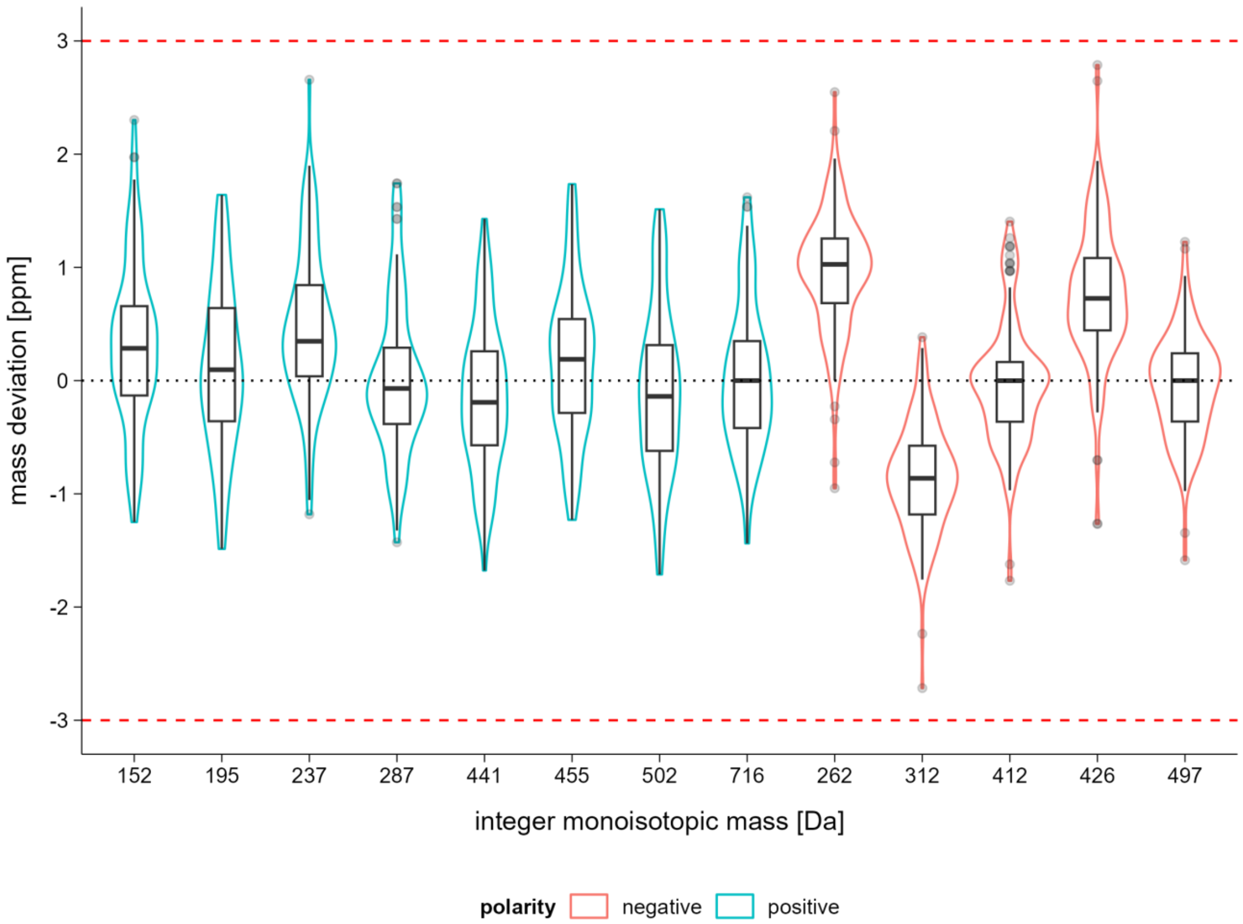

Figure S7: Mass deviation with only 2 injections for negative and positive mode. Acceptable threshold criteria in red, median displayed as black line.
